# Potential for gene silencing by piwi-interacting RNA in three lepidopterans

**DOI:** 10.3389/finsc.2026.1869174

**Published:** 2026-08-10

**Authors:** Santosh Poudel, Kevin Quito, Mosharrof Mondal, Kiran Nawaz, William Benjamin Walker, Alex Sutton Flynt

**Affiliations:** 1 Department of Biomedical Engineering, University of Mississippi, University, MS, United States; 2 Cellular and Molecular Biology, University of Southern Mississippi, Hattiesburg, MS, United States; 3 RNAissance AG LLC, St. Louis, MO, United States; 4 Temperate Tree Fruit and Vegetable Research Unit, United States Department of Agriculture - Agricultural Research Service (USDA-ARS), Wapato, WA, United States

**Keywords:** biopesticides, gene silencing, G-quadruplex, piwi-interaction RNA (piRNA), RNA interference (RNAi)

## Abstract

RNA interference (RNAi) has emerged as a promising strategy for insect pest control, yet its application in lepidopterans is hindered by insensitivity to double-stranded RNA (dsRNA). Here, we investigate the piwi-interacting RNA (piRNA) pathway as an alternative, mechanistically distinct gene silencing system in *Spodoptera frugiperda*, *Plutella xylostella*, and *Cydia pomonella*. Small RNA profiling revealed that piRNAs (26–30nt) are the predominant class across all species, marked by strong ping-pong signatures and extensive genomic distribution. Notably, abundant somatic expression, including in gut tissues, indicates that ubiquitous, endogenous piRNA populations may be able to initiate processing of exogenous RNA substrates. To test this possibility, synthetic piRNA-trigger constructs were found to induce gene silencing in Sf9 cells. Further, this approach to gene silencing offers opportunities for nucleic acid engineering, which we demonstrate by incorporating G-quadruplex structures that can enhance RNA stability. Together, these findings establish piRNAs as the dominant small RNA pathway in these lepidopterans and provide a mechanistic and design framework for next-generation RNA-based biopesticides.

## Introduction

RNA interference (RNAi) is a gene regulatory mechanism that silences genes at the post-transcriptional level and is guided by binding of small regulatory RNA molecules to target transcripts (1, 2). It is also the basis of established genetic technology where gene expression is controlled by small RNAs processed from an artificial precursor (1, 3). RNAi technology has many applications; one of which is in agriculture where RNAi is used to protect crops against arthropod pests through silencing essential genes (4). In current pest control applications, double-stranded RNA (dsRNA) is applied by spraying RNA solution to foliage or expressed via transgenes (5, 6). In this application, small RNAs called small-interfering RNAs (siRNAs) are the small RNA class intended to be produced after uptake by pest cells. The most overt biological function of siRNAs is as an antiviral response where their production from viral transcripts limits further replication (7). Arthropod genomes also encode a heterogeneous collection of endogenous siRNAs (endo-siRNAs) (8, 9). Universally, siRNAs are 20–23 nucleotides (nt) long and generated from >100nt dsRNAs that are processed by Dicer and then loaded into an Argonaute protein. In arthropods there are siRNA-specific Dicer (siDicer) and Ago (siAgo) proteins, which were first discovered in *Drosophila* as dme-Dicer2 and dme-Ago2 (10). Targeting by siAgo typically occurs with extensive complementarity to its target which invokes endonucleolytic cleavage or “slicing” of the target by the siAgo, leading to the target RNA’s destruction (11–13).

RNAi-based biopesticides have been commercialized for only a handful of coleopterans (beetles) and the varroa mite pests (14). This is due to significant limitations found with other arthropod species, especially lepidopterans, where exogenous dsRNA often produces inconsistent or weak silencing, largely due to dsRNA degradation and cellular barriers to uptake (15). Factors contributing to species-specific sensitivity to dsRNA include the presence of digestive enzymes that degrade dsRNA (dsRNases), variations in RNAi pathway component expression, and differences in gut permeability (16, 17). Indeed, the midgut environment of many Lepidopteran pests presents a major barrier to oral RNAi due to high dsRNA-degrading nuclease activity and physiological conditions that destabilize exogenous dsRNA, limiting the amount of intact RNA available for uptake. Furthermore, the high alkalinity of midgut in many insects can further accelerate dsRNA degradation (18–20). These challenges underscore the need for novel approaches to enable RNAi biopesticides.

One aspect of RNAi-mediated gene silencing that offers opportunities for innovation is in the type of small RNA produced from engineered transcripts (21–24). One such option is exploiting piwi-interacting RNA (piRNA)-mediated gene silencing instead of siRNA. Initially, piRNAs were assigned to a gonad exclusive function; however, it was later found that most arthropods express piRNAs in somatic tissue (25, 26). Work in hemipteran pests found that piRNAs could be used to suppress gene expression after ingestion of synthetic RNAs that “trigger” synthesis of piRNAs (27). Suppression of target transcripts by piRNAs was found to be comparatively effective as siRNAs.

piRNAs are produced through different molecular pathways than siRNAs. Unlike siRNAs, piRNA are generated through a Dicer-independent pathway and load into distinct Piwi clade Ago proteins (28, 29). Instead; piRNAs are produced from transcripts cleaved by Piwi-mediated slicing guided by a pool of pre-existing piRNAs (30, 31). The piRNAs that direct production of new piRNAs originate from multiple origins that include extranuclear inheritance, expression from dedicated piRNA clusters, and genic transcripts (32). Biogenesis of piRNAs is characterized by two processes: the ping-pong cycle and phased processing, which are carried out by partner Piwi proteins that we will refer to as phasing Piwi (phPiwi) and responder Piwi (rePiwi) (33–35). In phased processing, single-stranded precursor RNAs are processed into piRNAs by Zucchini endonuclease that load into phPiwi (36–38). Phasing is initiated by rePiwi cleavage guided by the sequence of a responder piRNA (33, 39). The ping-pong cycle operates under the same principle as phasing in that existing piRNAs trigger the mechanism. Here, after phPiwi cleaves a target RNA, its fragments are processed into responder piRNAs and loaded into rePiwi. In turn, cleavage products of rePiwi load into phPiwi to complete the cycle (26, 38).

The small piRNA molecules produced by these biogenesis pathways have several features that distinguish them from Dicer processed siRNAs and microRNAs (miRNAs) (39, 40). piRNAs are typically 26-30nt, making them longer than other small RNA classes. phPiwi loaded piRNAs usually have a 5’ uridine (1U), and if produced by phasing, can be found to align head to tail in a “phasing” pattern to their genomic origin. Another distinguishing signature is that ping-pong produced piRNAs have a 10-nucleotide overlap between pairs due to reciprocal slicing by phPiwi and rePiwi. Furthermore; as phPiwi-bound piRNAs have 1U bias rePiwi-bound piRNAs are enriched for adenine at position 10 (38, 41–43). Another hallmark of piRNAs is they often arise from genomic piRNA “clusters” that are rich in repetitive sequences, such as transposons (44, 45). This is due to the primary function of piRNAs in maintaining genome integrity (46–50). However, piRNAs do appear to have roles in control of protein coding gene regulation and can be produced from 3’ UTRs (51, 52).

The generation of ectopic piRNAs relies on several design guidelines (27, 53–55). The artificial transcript must contain a sequence complementary to species-extant piRNAs, specifically complementary to an abundant responder piRNA. This “piRNA trigger” segment can then be placed upstream of a target gene sequence so that when phasing is initiated; piRNAs are generated from the target. For this reason, the target gene sequence should be in the antisense orientation so that the generated piRNAs are complementary to the target. Further, the triggering RNA can be either single-stranded or double-stranded indicating that molecule’s structure is not essential to production of piRNAs. (26, 27, 37, 38).

In this work; we will explore the potential for piRNAs to induce gene silencing in three lepidopteran species: Codling Moth (*Cydia pomonella*, C. 56), Fall Armyworm (*Spodoptera frugiperda*, J.E. Smith, 57), and Diamondback Moth (*Plutella xylostella*, C. 56). These species represent some of the most challenging pests to control due to widespread insecticide resistance (58–60). These challenges intensify interest in alternatives such as RNA interference (RNAi); however, dsRNA based RNAi in these species has been reported to be inefficient (61, 62). As piRNAs can be produced from structurally diverse substrates; they may offer greater design options that are responsive to the specific biology of a target animal. To understand the potential for such an approach, we sought to annotate piRNAs in these species to explore the potential to design piRNA trigger sequences. This effort found that all species have abundant piRNAs in somatic tissues, including gut. Using an abundant piRNA generating sequence identified in *S. frugiperda;* we show that upon introduction of piRNA triggers in Sf9 cells gene silencing occurs. We also demonstrate the extensibility of piRNA-based gene silencing by testing terminal motifs for their ability to increase thermodynamic stability or interfere with exonuclease activity. The first two are the GNRA and UNCG tetraloops, which form highly stable RNA hairpin loops due to base-stacking and hydrogen-bonding. (**Supplementary Figures 3A, B**) (63–65). Next was a polyG that can fold into G-quartet structures (**Supplementary Figure 3C**) (66, 67). Another comes from a portion of the West Nile Virus Kunjin strain xrn1-resistant RNA 1 (Vxr), a structurally stable RNA element found in the 3’UTR of the WNV genome that has been shown to block exonucleases such as XRN1 which enhances its pathogenicity (**Supplementary Figure 3D**) (68, 69). The last one was a G-quadruplex (Gquad) structure that stacks three G-quartets (**Supplementary Figure 2E**) (70–72). Introduction of G-quad, more than other motifs, offered greater stability that led to enhancement of piRNA-mediated gene silencing, placing it on par with siRNAs in cell culture.

## Results

### Small RNA pathways and populations of three lepidopterans

To investigate the potential for ectopic piRNA expression in the three lepidopterans; we identified putative Ago/Piwi proteins in the genomes of, *S. frugiperda*, *P. xylostella, and C. pomonella* (**Supplementary Figure 1**) (73–75). Comparing these to homologs from *Drosophila melanogaster* and *Bombyx mori;* we found four Ago/Piwi homologs in each of the lepidopterans. Phylogenetic analysis revealed one homolog in *S. frugiperda*, two in *C. pomonella*, and two in *P. xylostella* clustered with Ago family, while the remaining two to three homologs fell within the Piwi clade. These results support the idea of conservation of both miRNA, siRNA and piRNA pathway components in Lepidopteran species, similar to what is reported in other arthropods (76).

With the presence of conserved small RNA pathway components, we next examined small RNA populations from multiple tissues and developmental stages. For *S. frugiperda*, nine libraries were generated, six from whole body tissues and three from guts. For *P. xylostella*, a large dataset was generated, consisting of 30 libraries derived from whole bodies (7 libraries), guts (20 libraries) and hemolymph (3 libraries). In *C. pomonella*, four libraries were constructed from two larval stages (3^rd^ instar and 5^th^ instar) with a sample from anterior and posterior of each stage. In total, sequencing datasets comprised 118 million reads for *S. frugiperda*, 646.4 million reads from *P. xylostella*, and 73.3 million reads for *C. pomonella*.

**Figure 1:**
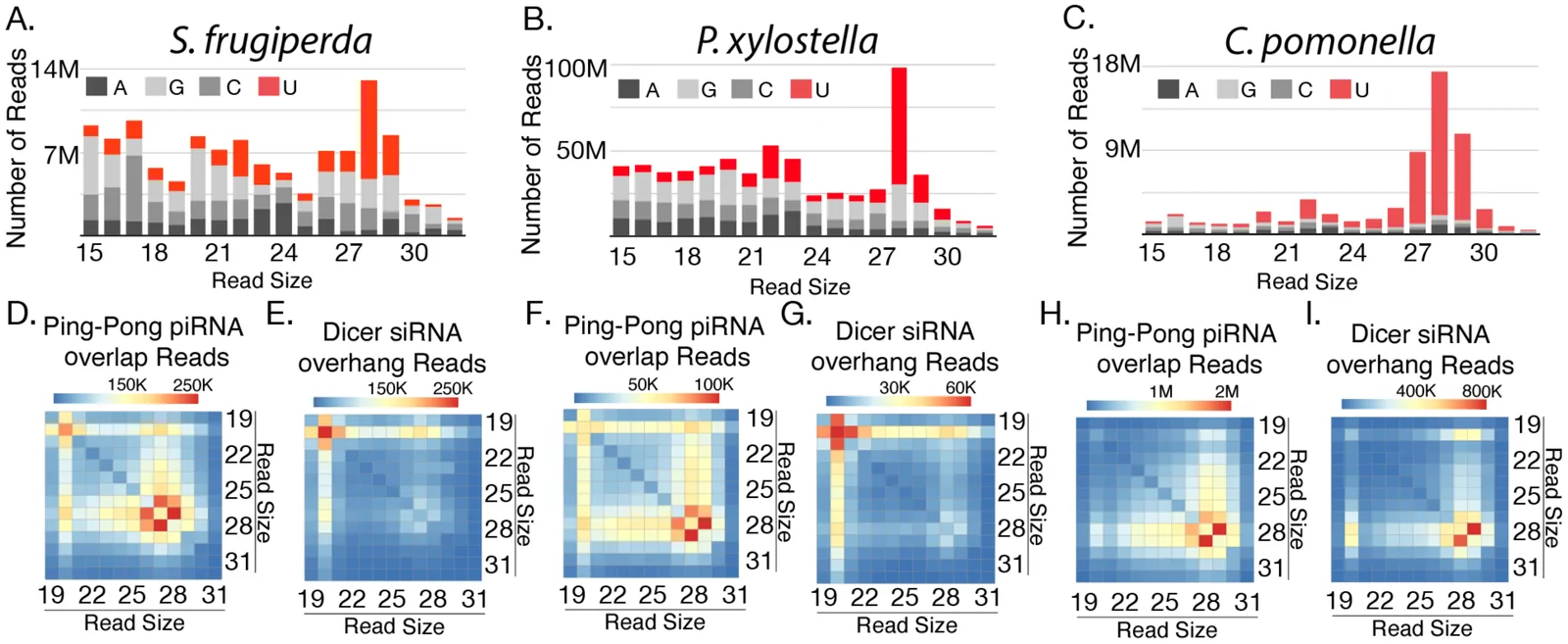
Small RNA size distribution and overall biogenesis signatures across three lepidopteran species. **(A–C)** Length distribution of total small RNA reads (15–32nt) mapped to the host genomes of *S. frugiperda*, *P. xylostella*, and *C. pomonella*. Colors in bar graph show the first base of the small RNA read. **(D–I)** Length-by-length overlap heatmaps showing small RNA pair biogenesis signatures. **(D, F, H)** Quantification of reads overlapping by 10nt as a measure of ping-pong biogenesis. **(E, G, I)** Quantification of 2nt overhangs to measure Dicer processing.

Reads were filtered to retain sequences between 15-32nt and mapped to their respective species’ genomes, resulting in mapping rates of 97.78%, 92.91%, and 97.03% for *S. frugiperda*, *P. xylostella*, and *C. pomonella*, respectively. To characterize small RNA populations, we first examined the distribution of read lengths (**Figures 1A, B**). All three species exhibited a pronounced peak at 28nt; corresponding to the size typical of piRNAs, with many fewer RNAs in the 20-23nt range corresponding to Dicer-derived small RNAs (miRNAs and siRNAs). Identification of first nucleotide identity revealed a strong bias for uridine at the 5′ end (1U bias) in the abundant, longer reads. Together, this indicates that piRNA-sized small RNAs represent the dominant class in all three species.

To investigate biogenesis mechanisms of the small RNA populations, we analyzed biogenesis signatures by quantifying alignments of small RNA pairs that are characteristic of ping-pong and Dicer processing (**Figures 1D–I**). Ping-pong biogenesis was assessed by quantifying reads that overlap by 10nt while Dicer processing by measuring reads that overhang by 2nt. In all three species, a prominent ping-pong signal was observed for reads 26-28nt (**Figures 1D, F, H**). siRNA-associated overlaps showed enrichment centered on ~20nt read pairs, corresponding to a 2-nt 3′ overhang, for *S. fruigperda* and *P. xylostella* (**Figures 1E, G**). This was not observed for *C. pomonella*, which showed most Dicer overhanging reads to be 27-29nt (**Figure 1I**). This unexpected result is likely a consequence of the incredible abundance of small RNA reads in the 27-29nt range found in *C. pomonella*. In that species the Dicer overhang analysis does show greater overlaps of 20nt reads with 27-29nt reads relative to ping-pong overlaps, suggesting that reads in the range of Dicer products tend to have 2nt overhangs. Further, 7% of all Dicer overhang reads are 20nt pairs, while 5% are 20nt pairs with a ping-pong signature. Together, these analyses show that while all species have both Dicer products and piRNAs, piRNAs are the dominant mode. This varies by species with *C. pomonella* having a notably more prominent piRNA population that significantly overlaps with potential Dicer products and is distinct from the *S. frugiperda* and *P. xylostella*.

### Characterization of lepidopteran small RNA processing

To further understand small RNAs in these animals, we characterized processing at individual genomic loci that are sources of these transcripts. Using mapped read alignments, small RNA producing loci were identified that excluded miRNA precursor loci based on size, to focus on siRNA and piRNA producing regions. Using this approach, we identified 143,688 small RNA expressing loci in *S. frugiperda*, 22,475 in *P. xylostella*, and 59,069 in *C. pomonella*.

**Figure 2:**
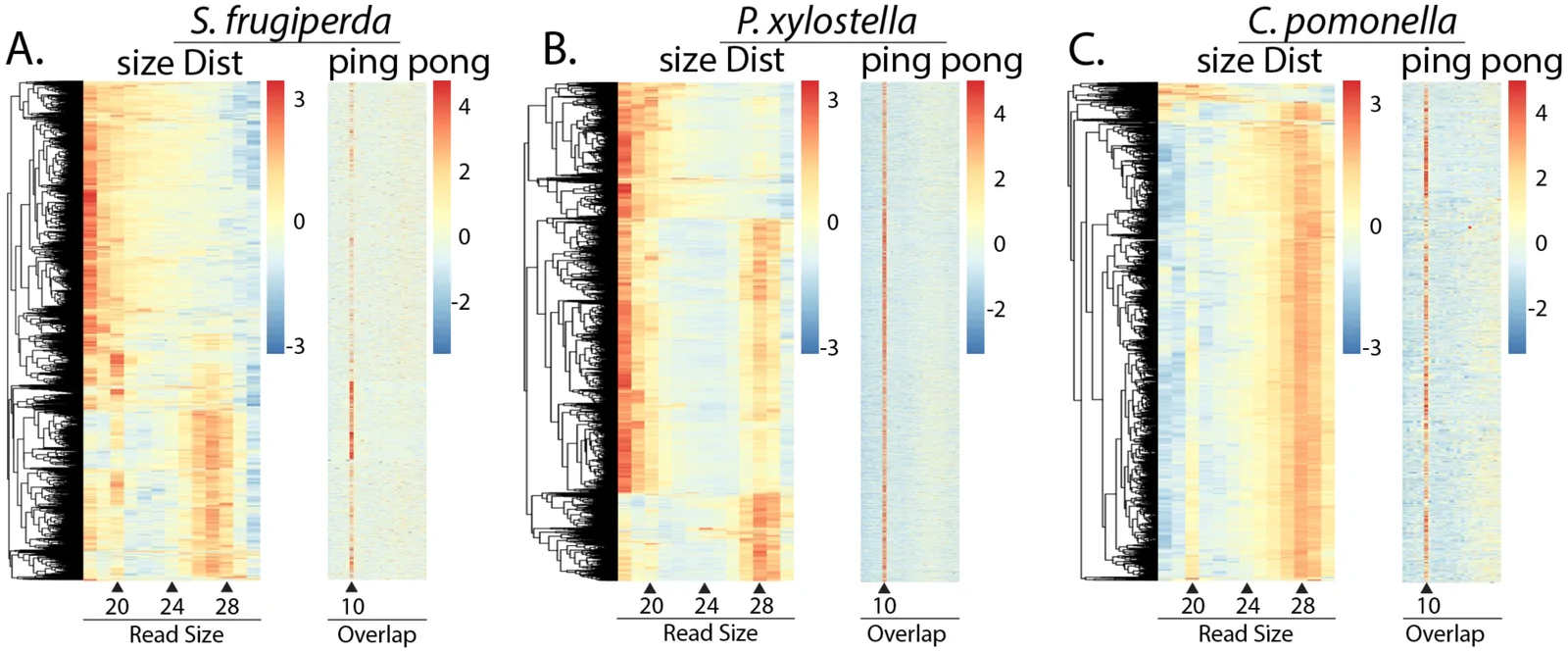
Small RNA locus size distribution and ping-pong signatures across individual small RNA–producing loci. Heatmaps showing read length distributions of locus (left panels) and overlap signatures (right panels) for small RNA–producing loci in **(A)** *S. frugiperda*, **(B)** *P. xylostella*, and **(C)** *C. pomonella*. Color key to right of all panels show Z-scores.

Next, we focused on the most abundant small RNAs, i.e. those that would be appropriate to develop into gene silencing technology, we analyzed read length distributions from the top 10,000 loci ranked by number of read alignments (**Figure 2**). To accompany assessment of read lengths we also examined overlap biases in alignments at each of the loci, which can reveal ping-pong processing if alignments show enrichment for 10nt overlaps. In *S. frugiperda;* roughly 40% of regions have a clear signal for piRNA sized reads (**Figure 2A**). However, even more of the loci exhibit ping-pong processing. The ping-pong signal at loci that do not show bias towards piRNA read size may be due to fragmentation of RNA where small (18-19nt) degradation fragments are the most common aligning read obscuring the relative signal associated with longer read alignment. Indeed; the *S. frugiperda* libraries have many smaller fragments (**Figure 1A**). *P. xylostella* showed an even greater portion of loci that had piRNA sized reads (**Figure 2B**). Similarly, there is even a more ubiquitous ping-pong signal in *P. xylostella* overlaps. *C. pomonella* had an even more loci (>90%) with clear piRNA sized reads, which is mirrored by a consistent ping-pong signature. This is consistent with the overall size distribution from *C. pomonella* where 27-29nt RNAs dwarf all other ranges (**Figure 1C**). Interestingly, there is also a clear peak of 20nt for many loci, suggesting the co-occurrence of Dicer products at these regions. This may explain the longer reads that exhibit 2nt overhangs suggestive of Dicer processing (**Figure 1I**). Together; these results show not only are piRNAs the most abundant small RNA in these species, but that they are also expressed from thousands of distinct loci, suggesting widespread regulation by the piRNA pathway.

### piRNA expression in different tissues

**Figure 3:**
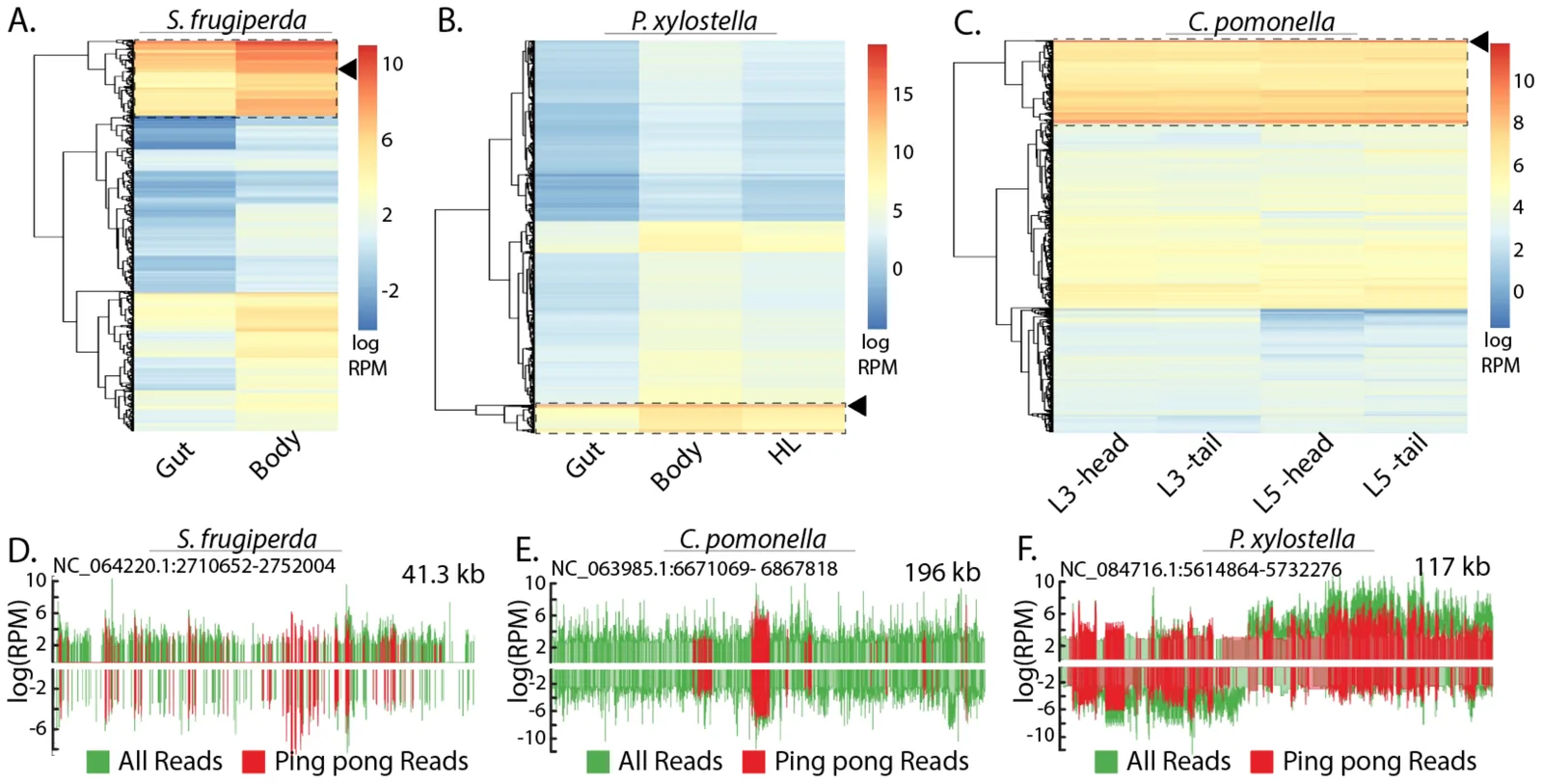
Tissue-specific expression of piRNAs. **(A–C)** Heatmaps showing the abundance of piRNA-sized reads (24–32nt; log RPM) across small RNA–producing loci in different tissues. **(D–F)** Sushiplot of representative loci appropriate for design of piRNA triggers. Small RNA read coverage for all reads in green and reads with ping-pong signature in red.

piRNAs are reported to be tissue specific in many species (26). Exploiting piRNAs for controlling gene expression requires endogenous piRNAs to initiate processing of exogenous RNAs (27, 77). To examine the suitability of specific piRNAs for inducing gene silencing we quantified expression of piRNAs generated from genomic regions in different tissues. Regions of interest were identified as those that had a bias towards longer (27-30nt) reads that also have ping-pong alignments (**Figures 3A–C**). The abundance of piRNA-sized reads (26–30nt) was quantified from each region and normalized as reads per million (RPM). In *S. frugiperda*, piRNA expression was compared between gut and whole-body samples (**Figure 3A**). In *P. xylostella*, gut, whole body, and hemolymph was contrasted (**Figure 3B**). For *C. pomonella*, anterior and posterior samples are compared from third and fifth instar larva (**Figure 3C**). These data show that somatic tissues contain significant populations of piRNAs by comparing soma only samples to whole body or posterior samples containing gonad tissues. Gonad is often marked by high levels of piRNA production (44). Importantly, significant piRNA expression is seen in gut samples where ingested RNAs that trigger piRNA production would need to be active. While there is variability in expression between tissues, which is pronounced in *S. frugiperda*, there is a set of highly expressed piRNA loci that are consistently producing piRNAs across samples. This indicates that despite variability in piRNA expression there are loci, in all three species, that can be exploited for gene silencing in all tissues surveyed. Examples of piRNA loci that are expressed consistently across tissues show stereotypical features of dual strand piRNA clusters with reads with ping-pong overlaps and many apparent phasing-type piRNAs (**Figures 3D–F**). This suggests that strategies for generating RNAs reported in *B. tabaci*, the whitefly, should be appropriate for these lepidopterans (27).

### Efficient piRNA trigger mediated gene silencing in *S. frugiperda* cells

**Figure 4:**
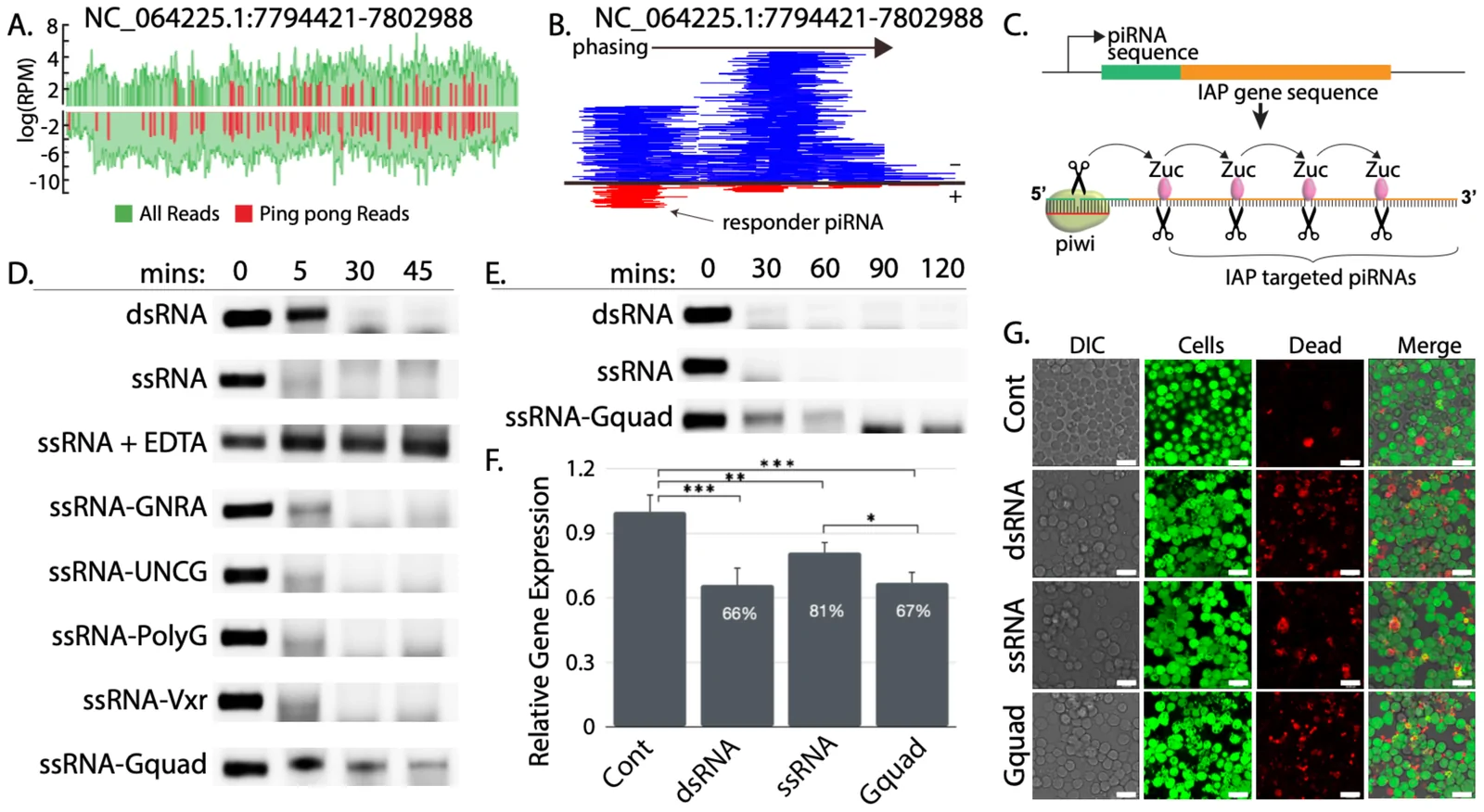
Identification and functional validation of a piRNA-triggering system for gene silencing. **(A)** Sushiplot of a highly expressed piRNA-producing locus in Sf9 cells (NC_064225.1), showing total small RNA reads (green) and ping-pong pairs (red), used to design a piRNA trigger. **(B)** Detailed view of piRNA trigger sequence showing the area of responder piRNAs along with phased piRNA in each end. Reads are colored by plus strand (red) and minus strand (blue) **(C)** Schematic of the piRNA-triggering mechanism. **(D, E)** Stability assays of RNA molecules incubated in gastric fluid. **(F)** Quantification of gene silencing by RT-qPCR following transfection of Sf9 cells with dsRNA, ssRNA, or Gquad-stabilized ssRNA triggers. **(G)** Live/dead cell imaging following treatment shows increased cytotoxicity in cells treated with dsRNA and Gquad-stabilized ssRNA compared to controls and unmodified ssRNA. The statistical significance symbols mean: *P < 0.05, **P < 0.01, and ***P < 0.001.

To examine the potential for piRNA-mediated gene silencing in lepidopterans; we sought to elicit this mode in Sf9 cells. Gut and Sf9 cells appear to possess similar piRNA biogenesis mechanisms with both ping pong and phasing type processing being present, suggesting that if piRNA-mediated silencing can be elicited in Sf9 cells the same processes will be functional in gut cells (**Supplementary Figure 2**). To achieve this, we sought a piRNA trigger sequence that is cognate to gut and Sf9 cell piRNAs. To identify such a loci, regions analyzed above (**Figure 3A**) were examined for expression in gut libraries and from publicly available sequencing data from Sf9 cells (78). Among the identified loci, NC_064225.1:7794421–7802988 exhibited the highest read density, with approximately 2.1 million reads in the Sf9 libraries. Within this region, responder piRNAs were identified at coordinates NC_064225.1:7798749–7798785 based on their characteristic length and the presence of a 10A, which is a signal for Piwi-mediated slicing directed by a 1U piRNA. These observations, together with the presence of phased piRNAs at both ends of the locus, supported the selection of this region as the source for the trigger sequence. (**Figures 4A, B**). This piRNA trigger was then fused to a sequence complementary to the *S. frugiperda* Inhibitor of apoptosis (IAP) gene. When cleaved by an endogenous responder piRNA from the NC_064225.1:7798749–7798785 locus, phasing piRNAs should be generated that can direct cleavage of this target (**Figure 4C**). The target gene was chosen due to knockdown of IAP expression leading to disinhibition of apoptosis and creating measurable cellular phenotypes.

A major concern with RNA molecules for pest control is stability, and single-stranded (ssRNA) molecules such as the synthetic piRNA trigger investigated here can be expected to be less stable than dsRNA (79). To enhance the stability of a prospective piRNA trigger we tested five prospective RNA stabilizing motifs (GNRA, UNCG, PolyG, WNVKUN-xrRNA1, and Gquad) (**Supplementary Figure 3**). The ability of the elements to stabilize synthetic RNAs was tested by monitoring full-length RNA abundance after incubation in gastric fluid collected from 4th and 5th instar *S. frugiperda* larva over 45 mins (**Figure 4D**). The results confirm the greater instability of ssRNA relative to dsRNA with degradation fragments in some time points. This effect is dependent on nucleases which can be inactivated by addition of EDTA. While several motifs such as GNRA provided modest increases in stability, only the Gquad structure conferred substantial enhancement, showing greater stability than dsRNA. Next, we compared the stability of Gquad RNAs to ssRNA and dsRNA over a longer incubation time – up to 120 mins (**Figure 4E**). Here we found Gquad RNAs persisting up to an hour in gastric fluids, offering substantially more stability than dsRNA which is nearly entirely lost after a 30-minute incubation.

To evaluate the efficacy of piRNA-based triggers in inducing gene silencing, Sf9 cells were transfected using Lipofectamine with dsRNA targeting GFP (Control), a dsRNA version of the piRNA trigger, an ssRNA trigger without stabilizing motifs, or Gquad added ssRNA trigger (**Figure 4F**). After 24 hours, qPCR was used to measure expression of IAP, showing equal level gene silencing by dsRNA (66%) and Gquad ssRNA (67%). While the basic ssRNA achieved a more modest reduction in IAP transcript levels (81%) it was still significant relative to control. Notably, the difference between the destabilized and Gquad-stabilized ssRNA treatments was statistically significant, highlighting the capacity of structural modifications to enhance RNAi efficacy. These changes in gene expression were supported by cellular phenotypes (**Figure 4G**). Live-cell imaging showed minimal Ethidium homodimer (dead-cell stain) fluorescence in GFP-treated control group. In contrast, cells treated with the dsRNA trigger and the Gquad ssRNA exhibited substantial increases in the number of dead cells (**Supplementary Figure 4**). The unmodified ssRNA trigger led to an increase in dead-cell signal relative to control, though not as significant as dsRNA or Gquad, consistent with less efficiency of gene silencing.

To further investigate the behavior of the piRNA trigger, small RNA sequencing was performed on cells transfected with the Gquad version of the trigger (**Supplementary Figure 5**). Alignment of reads to the sequence of the synthetic RNA found over a quarter of a million reads mapping that are piRNA sized (27-30nt). Alignments occurred along the entire length of the sequencing and importantly occurred in the IAP portion of the RNA. This contrasted with sequencing from untreated cells where no IAP targeted RNAs were found. Importantly, we found reads mapping to the junction between the piRNA segment and IAP segment. The sequence of the of this site is unique to the trigger and does not appear in the *S. frugiperda* genome, providing definitive proof that the synthetic RNAs are processed into fragments that possess features of piRNAs.

## Discussion

This study provides comprehensive overview of endogenous small RNAs in three economically important lepidopteran pests *S. frugiperda*, *P. xylostella*, and *C. pomonella* with the goal of informing RNAi-based pest control strategies. Lepidopterans are generally regarded as insensitive to next generation RNAi biopesticides based on dsRNA-mediated gene silencing. Advancing this strategy will require alternative approaches that may extend to the RNA molecules used to trigger gene silencing. To inform such a novel approach, we performed small RNA profiling to understand the dominant modes of endogenous gene silencing in the three pests. From sequencing nearly 850 million small RNAs across the three species; it was clear the dominant class of small RNA is piRNAs, regardless of tissue type. This contrasts with other arthropods where siRNA-class small RNAs are the most abundant type (80–82). Surveying small RNA generating loci revealed tens of thousands of individual sites that generate piRNA furthering showing the widespread nature of regulation by this class in these pests, which has been seen in other organisms (25, 53).

While all insects in the study showed highly abundant piRNA expression; there were some clear species-specific biology. This was most apparent when comparing *C. pomonella* to the other two species. The codling moth showed an order of magnitude greater bias towards piRNAs production, based on comparing the size distribution to what was found in other species. Most unexpectedly, the observation of longer short RNAs (27-29nt) with Dicer overhang signatures, suggests a potential intertwining of siRNA and piRNA pathways that might be specific to this animal. Similar observations have been observed in other arthropods, but this would be a case of convergent evolution (83, 84). It also highlights the need to investigate small RNA biogenesis on a case-by-case basis to assist with rationale design of gene silencing approaches.

Considering the apparent importance of piRNAs to gene regulation in these species, this led us to explore using this pathway for gene silencing in lepidopterans. This approach with piRNA was previously found to be effective in the hemipteran pest, *B. tabaci* (whitefly) where piRNA-mediated gene silencing occurred after feeding of synthetic piRNA triggers (27). Ectopic piRNA production in *B. tabaci* led to specific gene silencing effects, even outperforming siRNA triggers. Here we sought to validate this approach in Sf9 cells using lipofectamine transfection to determine if delivery of piRNA triggers can induce gene silencing. From this test we found that these molecules can be effective in silencing gene expression.

In order to reach the same level of gene silencing relative to dsRNA in this assay, increased stability of the trigger RNA was needed. Using a gastric fluid-based digestion assay, multiple prospective stabilizing motifs were tested, revealing that Gquad elements are sufficient to enhance stability. The Gquad sequences likely have a greater impact due to formation of multiple G-quartet structures (**Supplementary Figure 3**). Gratifyingly, these sequences provide even greater stability than dsRNA and could achieve a similar level of gene silencing as dsRNA. This highlights an important feature offered by piRNA-based gene silencing. Unlike dsRNAs, which must fold into a-form helices that have few features that distinguish different sequences, piRNA triggers do not have a strict molecular structure. siDicers recognize dsRNA structure, requiring ends that can be recognized by Dicer PAZ domain (85, 86). Piwi proteins, on the other hand, recognize sequences not structure. This offers the possibility for additional engineering. Motifs could be added that not only affect stability but could help to guide intercellular trafficking, bestow cell penetrating functionality, or sequences that can associate with nanocarrier structures. Together this work suggests that RNAi design should be guided by knowledge of endogenous small RNAs and that alternatives to standard approaches exist that may enable new possibilities for RNAi-based biopesticides.

## Methods

### Tissue collection and small RNA sequencing

*S. frugiperda* and *P. xylostella* larvae were obtained from Benzon Research (Carlisle, PA, USA) and maintained on artificial diet (Southland Products, Lake Village, AR, USA) under controlled environmental conditions (26 °C, 50% relative humidity, and a 16:8 h light:dark photoperiod). *C. pomonella* larvae were reared in-house on artificial diet under identical environmental conditions at the USDA-ARS Temperate Tree Fruit and Vegetable Research Unit (Wapato, WA, USA). For *C. pomonella*, newly eclosed adult females were paired with males and allowed to mate and oviposit, and neonate larvae were transferred to 128-well bioassay trays containing artificial diet for development (87, 88).

Larvae were collected at defined developmental stages for each species. For *C. pomonella*, third and fifth instar larvae were harvested at 7 and 14 days post-hatching, respectively. For *S. frugiperda* and *P. xylostella*, second instar (L2) larvae were collected. Tissue-specific dissections were performed for each species: head and tail segments for *C. pomonella*; whole body and gut tissues for *S. frugiperda*; and carcass, gut, and hemolymph fractions for *P. xylostella*. All dissections were carried out using sterile tools, and tissues were immediately transferred into 1.5 mL microcentrifuge tubes containing RNAlater (Thermo Fisher Scientific, Waltham, MA, USA) to preserve RNA integrity.

Total RNA was extracted using the PureLink miRNA Isolation Kit (Invitrogen, Thermo Fisher Scientific) according to the manufacturer’s protocol and eluted in RNase-free water. RNA quality and concentration were assessed prior to library preparation. Small RNA libraries for *C. pomonella* samples were prepared at Maryland Genomics (Baltimore, MD, USA) using the NEXTFLEX^®^ Small RNA-Seq Kit v3 (PerkinElmer, Austin, TX, USA), with diluted adapters and gel-free size selection using AMPure SPRI-select beads. Libraries were amplified and assessed for fragment size and concentration prior to sequencing. Libraries for *S. frugiperda* and *P. xylostella* were prepared at the University of Mississippi Medical Center (UMMC) core facility using the NEXTFLEX^®^ Small RNA Sequencing Kit v4 (PerkinElmer) following the manufacturer’s protocol. All libraries were pooled and sequenced on an Illumina NovaSeq 6000 platform using an S4 flow cell, targeting approximately 10 million reads per sample. All clipped data are available through the NCBI SRA database under the bio project #PRJNA1457568.

### Phylogenetic tree construction

Amino acid sequences of Argonaute (AGO) proteins from *Drosophila melanogaster* and *Bombyx mori* were retrieved from the NCBI protein database. Genome assemblies and annotation files for *S. frugiperda* (AGI-APGP_CSIRO_Sfru_2.0), *P. xylostella* (ilPluXylo3.1), and *C. pomonella* (ilCydPomo1) were downloaded from NCBI.

Putative AGO sequences were curated using BLASTp and confirmed via conserved domain search (NCBI CDD and Pfam). Multiple sequence alignment was performed using MUSCLE, and a phylogenetic tree was constructed using the Maximum Likelihood method through Phylogeny.fr (89). Tree visualization was done using TreeDyn.

### Small RNA analysis and bioinformatics pipeline

Raw sequencing data quality was assessed using FastQC. Adaptor-trimmed reads between 15–32nt were mapped to their respective genomes using Bowtie (v1.2.3). Regions of small RNA expression were defined as genomic loci with read depth greater than 10 and merged if located within 500 nucleotides of each other. Loci shorter than 100 bp were removed to generate a collection of non-miRNA type small RNA expressing loci. Mapped reads were processed using SAMtools and BEDTools to quantify read alignment and identify high-expression regions (**Supplementary Figure 6**). Read counts were normalized to the total number of genome-mapped reads for each sample. piRNA biogenesis signatures: 10nt 5′ overlap between read pairs indicated the Ping-Pong signature and 2nt 3′ overhang between paired reads indicated Dicer processing using a small RNA analysis software (90). Data visualization was performed using R packages: ggplot2, pheatmap, and sushiplot (91–93).

### piRNA trigger design and synthesis

150nt of a piRNA locus was fused (**Figure 4A**) to a 124nt anti-sense sequence of *S. frugiperda IAP* protein 3 (XM_035602447.2). Derivatives of this construct were generated by plasmid synthesis by Genscript^©^. This construct was amplified by primers containing a T7 promoter sequence either on both the forward and reverse primers (for dsRNA synthesis) or only on the forward primer (for ssRNA synthesis). Following PCR and *in vitro* transcription, the resulting RNA was denatured by placing tubes into a heating block set to 90 °C for 2 minutes and then slowly reannealed by removing the block and allowing it to cool for 1 hour. RNA purity and concentration were assessed using a NanoDrop, after which the samples were aliquoted for convenient single-use applications.

### Sf9 transfection, imaging, and qPCR

Cells were obtained from Millipore Sigma and cultured in Grace’s media supplemented with 10% FBS, 1% PenStrep at 27 °C. For transfections, a 6-well plate was seeded with 2 mL of cells at a density of 1 × 10^6^ cells/mL in Grace’s medium lacking FBS and antibiotics. 5µg of RNA was complexed with Lipofectamine 3000 following manufacturer’s protocols and delivered to cells for either 24 hours or 48 hours. Following incubation, cells were harvested and RNA extracted using TRI reagent. One microgram of RNA was used as input for cDNA synthesis with ThermoFisher’s Maxima H Minus kit, and the resulting cDNA was diluted 1:2 before performing qPCR on a CFX-OPUS using Applied Biosystems™ SYBR™ Green Universal Master Mix. Primers used for qPCR were designed to amplify a region >100bp downstream of where the RNA triggers targeted (**Supplementary Table**). To measure cell death Sf9 cells were transfected using Lipofectamine and incubated for 24 hours prior to staining with the Invitrogen LIVE/DEAD imaging kit followed by confocal imaging with a Leica Stellaris confocal microscope.

### Gastric fluid collection

Gastric fluid was collected from 4th and 5th instar FAW larvae. Larvae were surface sterilized in 70% EtOH before having their midguts removed. Approximately 20 midguts were collected and 100µl of ice-cold 1x sterile PBS was added before being homogenized. The lysate was centrifuged for 15 minutes at max speed at 4 °C. The supernatant was collected and 100µl of 80% glycerol (1:1) was added. The gastric fluid was stored at -20 °C.

### RNA degradation assays

RNAs were diluted to 500ng/µl and 2µl (1µg) added to a sterile PCR tube on ice. Each reaction was brought to a volume of 9µl by adding nuclease-free water followed by brief vortexing (2–3 seconds) and spinning down the contents. 1 µl of midgut digestive extract was added to the tube, vortexed briefly and spun down again (total reaction volume = 10µl). Reactions were incubated at 26 °C. For subsequent gel electrophoresis, samples were run on a 1% TAE agarose gel at 125V for 25 minutes. Gels were imaged using a Bio-Rad Gel Doc Imaging System.

## Data Availability

The datasets presented in this study can be found in online repositories. The names of the repository/repositories and accession number(s) can be found in the article/**Supplementary Material**.
