# Supplementary material for: Potential for gene silencing by piwi-interacting RNA in three lepidopterans
Source: Front Insect Sci. 2026 Aug 10;6:1869174. doi: 10.3389/finsc.2026.1869174 (PMC13501171; doi:10.3389/finsc.2026.1869174)
Supplement: Supplementary file 1 [file SupplementaryFile1.docx]

**Supplemental Information**

**Phylogenetic Analysis of PIWI Proteins in 3 Lepidopteran Species**

**Supplemental Figure1.** Phylogenetic tree showing conservation of crucial PIWI proteins in three significant lepidopteran crop pests (*P. xylostella, S. frugiperda,* and *C. pomonella)*. This confirms the required cellular machinery exists in all 3 species to perform piRNA-mediated gene silencing.

**Supplemental Figure 2.** **Presence of ping pong processing and phasing biogenesis signatures in gut and sf9 sequencing data.** Each row of heatmap represents high expressing loci from the respective tissues. Triangles point to positions where signal is expected for each type. **A)** Bias in overlaps between reads mapping to piRNA loci in gut. Triangle points to 10nt overlap. **B)** Biases of distance from upstream read in gut. Triangles point to enrichment for close distances and to lack of signal that occurs due to the length of piRNA reads. **C)** Bias in overlaps between reads mapping to piRNA loci in sf9 library. Triangle points to 10nt overlap. **D)** Biases of distance from upstream read in sf9 sample. Triangles point to enrichment for close distances and to lack of signal that occurs due to the length of piRNA reads.

**Supplemental Figure 3. Structural RNA motifs used to enhance stability of synthetic piRNA triggers.** RNA secondary structures and sequence motifs were evaluated for their potential to enhance RNA stability and resistance to nuclease degradation. Secondary structures were predicted using RNAfold. A) GNRA tetraloop B) UNCG tetraloop C) Poly12 G D) WNV-Kun and E) G-quadruplex

**Supplemental Figure 4. Quantification of red (dead cell) signal**. ImageJ was used to quantify red and green signal within 5 uniform regions of every group and the bar chart shows the average proportion of red signal to total signal (Red/Red+Green). Error bars represent the standard deviation across the 5 red signal measurements for every group and a Student’s t-test was used to determine p-value relative to the negative control, GFP.

**Supplemental Figure 5.** **piRNA-sized** **reads aligning to the synthetic G-quad piRNA trigger sequence** in A) cells treated with the synthetic RNA and B) untreated cells. Reads colored by first base. Features of the sequence shown in cartoon below. Dashed box highlights the junction unique to the trigger – does not appear in the *S. frugiperda* genome.

**Supplemental Figure 6. Computational pipeline for size-based partitioning of small RNA reads. Filtered small RNA sequencing reads were divided into siRNA-sized (21–24 nt) and piRNA-sized (26–30 nt) populations prior to genome alignment and downstream overlap analysis.**

| **Primer** | **Sequence** |
| --- | --- |
| Gquad_FP | TAATACGACTCACTATAGGGTAAGGGCTAGGGTTAGGGTTTTGGGAGGACGATGC |
| Gquad_RP | GGGTAAGGGCTAGGGTTAGGGAAAAGCAGCCAGCTATGGGCTAAGAAAAGG |
| ssBasic_FP | TAATACGACTCACTATAGGGTGTTCGGGAGGTTCC |
| ssBasic_RP | TCCCGTGCACCCACGATATGC |
| dsBasic_RP | TAATACGACTCACTATAGGGTCCCGTGCACCCACGATATGC |
| PolyG _FP | TAATACGACTCACTATAGGGGGGGGGGGGGGGTTTTGGGAGGACGATGC |
| PolyG_ RP | CCCCCCCCCCCCAAAAGCAGCCAGCTATGGGCTAAGAAAAGG |
| WNV-kun-_xrRNA_FP | TAATACGACTCACTATAGGGAGTCAGGCCGGAAAATTCCCGCCACCGGAAGTTGAGTAGACGGTGCTGCCTGCGACTGGGGGGGGGGGGTTTTGGGAGGACGATGC |
| GNRA_FP | TAATACGACTCACTATAGGGTCGCGCAAGCGTTTTGGG |
| GNRA_RP | CGCUUGCGCGCGCTTGCGCGAAAAGCAGCCAGC |
| UNCG_FP | TAATACGACTCACTATAGGGCGCTACGGCGTTTTGGGAGGACGATGC |
| UNCG_RP | CGCCGTAGCGAAAAGCAGCCAGCTATGGGCTAAGAAAAGG |
| 18s_qPCR_FP | GACTCAACACGGGAAATCTC |
| 18s_qCPR_RP | CCACGCACACCTAAATGAC |

**Supplemental Table 1.** Primers were designed to incorporate the T7 promoter sequence (TAATCGACTCACTATAGGG) on the 5’ terminus for ssRNA products or both 5’ and 3’ for dsRNA products. Stability motifs were incorporated immediately downstream of the T7 promoter sequence on forward primers or terminally for reverse primers so that resulting RNA would be bookended by the motif.

**Poly-G knots:**

| **Motif** | **Sequence** |
| --- | --- |
| G-N-R-A | GCAA |
| U-N-C-G | TACG |
| PolyG tract | GGGGGGGGGGGGGGGGGG |
| WNVKUN-xrRNA1 | AGTCAGGCCGGAAAATTCCCGCCACCGGAAGTTGAGTAGACGGTGCTGCCTGCG  ACT |
| G-quadruplex | GGGCTAGGGTTAGGGTTTTGGG |

**Supplemental Table 2.** Names and sequences of candidate motifs that were tested in RNA degradation assays for their ability to confer nuclease resistance.
